# Long-Term Effects of a Multi-Component Community-Level Intervention to Reduce Single-Vehicle Nighttime Crashes: Follow-Up Findings From a 24-Community Randomized Trial

**DOI:** 10.15288/jsad.24-00103

**Published:** 2025-02-05

**Authors:** Robert Saltz, Mallie J. Paschall

**Affiliations:** ^a^ Prevention Research Center, Pacific Institute for Research & Evaluation, Berkeley, California

## Abstract

**Objective:**

This follow-up study examines whether a multi-component, high-visibility alcohol enforcement intervention implemented in 12 California cities had long-term effects on alcohol-related motor vehicle crashes beyond the period of the original study. Previous results indicated a significant reduction in single-vehicle night-time (SVN) crashes among 15- to 30-year-olds in intervention cities relative to controls.

**Method:**

A randomized trial was conducted with 24 randomly chosen California cities from 2012 to 2017 to evaluate a multi-component intervention to reduce excessive drinking and driving while impaired among adolescents and young adults. Twelve of the cities were randomly assigned to the intervention condition and implemented high-visibility alcohol enforcement operations and other components from April 2013 to March 2016. Multi-level negative binomial regression analyses were conducted with motor vehicle crash data from 2010 to 2021 to examine whether SVN crashes among 15- to 30-year-olds decreased in intervention cities relative to controls after the multi-component intervention was implemented. Analyses controlled for community sociodemographic characteristics, the overall time trend, the COVID pandemic, and pre-intervention levels of SVN crashes and adjusted for correlation of repeated observations within cities over time.

**Results:**

Regression analyses indicated a significantly lower level of monthly SVN crashes among 15- to 30-year-olds in intervention cities during post-intervention months through 2021 relative to control cities (incidence rate ratio [95% CI] = 0.88 [0.79, 0.98], *p* < .05) when controlling for community sociodemographic characteristics, the overall time trend, COVID, and pre-intervention levels of SVN crashes.

**Conclusions:**

Study findings suggest that a multi-component, high-visibility alcohol enforcement intervention can have long-term effects on alcohol-related motor vehicle crashes and related injuries and fatalities among adolescent and young adult drivers.

Starting with a community-level prevention approach, Saltz and colleagues (2021), working in collaboration with the California Department of Health Care Services and funded by the federal Substance Abuse and Mental Health Services Administration (SAMHSA), designed a group-randomized trial involving 24 California cities with an intervention to reduce alcohol-related harm among 12-to 25-year-olds (as specified by the grant). Of note, the cities were selected at random, paired by demographic characteristics, and then assigned to treatment or control conditions at random as well. The multi-component intervention included enhanced and highly visible enforcement of driving-under-the-influence (DUI) laws and alcohol sales to minors, along with training in responsible beverage service. The study found an estimated 310 fewer alcohol-involved crashes in the intervention cities relative to controls over a 3-year period from the start of intervention implementation. Details are provided in Saltz et al. (2021).

This brief report takes advantage of additional years of crash data, extending from 2016 to 2021, to assess the possible long-term effects of the community intervention. At the same time, greater statistical power allows us to assess possible effects of the COVID-19 pandemic and include preintervention levels of single-vehicle nighttime (SVN) crash rates as an additional control in the analyses.

Federal funding (whether SAMHSA or National Institutes of Health) is enormously helpful in making such research and evaluation studies possible, but the time covered by those grants (typically 5 years) means that implementation must be achieved in 1 or 2 years so that postintervention data can be collected and analyzed within the funding period, an especially significant challenge when working at the community level. Even when successful, it leaves the question of whether the intervention effects continue beyond the first couple of postintervention years. Moreover, archival outcome data, such as California motor vehicle crash data, are often not available for up to 2 years after the crashes occur, further limiting opportunities to assess longer-term postintervention effects within the grant funding period.

The primary outcome measure used to evaluate the intervention was SVN crashes, a commonly used proxy for alcohol-involved motor vehicle crashes. These would comprise the most common cause of injuries and deaths among youth and young adults and were also a source of data that would not require overt original data collection at the comparison cities. This outcome measure also allows us to revisit the evaluation by extending the postintervention period. Would the reduction in crashes in comparison to control cities extend beyond the life of the project?

## Method

### Study design

We conducted a randomized controlled trial from 2012 to 2017 with 24 medium-sized cities (50,000–450,000 population) in California to evaluate high-visibility alcohol enforcement operations aimed at reducing excessive drinking, drinking and driving, and alcohol-related motor vehicle crashes among adolescents and young adults (Saltz et al., 2021). The research protocol was reviewed and approved by the Institutional Review Board of the Pacific Institute for Research & Evaluation.

### Intervention

After about a year of planning, the intervention period was from April 2013 through March 2016. Alcohol enforcement operations conducted by local enforcement agencies included DUI sobriety checkpoints and saturation patrols to reduce drink-driving and undercover operations in bars or other licensed on-premises establishments to reduce alcohol service to obviously intoxicated patrons. Visibility activities included publicizing local enforcement operations through various media channels (e.g., radio, social media, mobile signs, marquees at high schools), periodic bar visits by local enforcement officers, and letters to owners/managers of licensed on-premises establishments. In most intervention cities, a community-based organization (CBO) was also involved in publicizing enforcement operations in collaboration with local enforcement agencies. Enforcement agencies and CBOs were expected to implement at least two high-visibility enforcement operations per quarter.

### Measures

*Single-vehicle nighttime crashes*. We used data from the California Statewide Integrated Traffic Records System to create a monthly measure of SVN crashes for each city from January 2010 through December 2021 (3,456 observations) compared with an end date of March 2016 in the original article. Specifically, we determined the number of all motor vehicle crashes that were SVN crashes for 15- to 30-year-old drivers. SVN crashes are a commonly used and valid proxy for alcohol-related motor vehicle crashes when blood alcohol concentration data for drivers are not available, and assessment of driver impairment is only based on the judgment of law enforcement officers for non-injury and nonfatal crashes (Voas et al., 2009).

*Time, seasonal, and COVID-19 pandemic covariates*. A continuous time variable representing 144 months was created for analyses. Based on seasonal variation in SVN crashes, we included a dummy variable for early fall months (September/October) because SVN crash rates were consistently lowest in these months from 2010 to 2021. In addition, we included a COVID dummy variable corresponding to the period from March 2020 to December 2021 because SVN crashes increased during the pandemic.

*Intervention condition*. An intent-to-treat intervention condition variable was coded zero for all 3,456 months for the 12 control cities. For the 12 intervention cities, the intervention condition variable was coded zero only for pre-intervention months and was coded one for postintervention months (April 2013–December 2021).

*Intervention dosage*. Monthly intervention activities were reported by local enforcement agencies and CBOs from April 2013 through March 2016. The average number of monthly intervention activities specifically related to drink-driving was calculated for each city. Cities were then classified as meeting a minimum average threshold of two high-visibility DUI enforcement operations per quarter or not meeting this threshold over the 3-year period. Two dummy variables were created for intervention cities to represent two or more high-visibility DUI operations per quarter or less than two high-visibility DUI operations per quarter.

*City characteristics*. City demographic characteristics included population size, percentage of ethnic minorities, and a socioeconomic status factor score based on percentage of residents living below poverty, percentage unemployed, percentage with a college degree, and median household income. These characteristics are based on 2011 census data. Because the two groups of cities were non-equivalent in SVN crashes before the intervention period began, we created a city-level measure of SVN crashes as a percentage of total motor vehicle crashes for pre-intervention months to include as a covariate in analyses.

### Data analysis

Multi-level negative binomial regression analyses were conducted to examine whether the intervention had an effect on SVN crashes among 15- to 30-year-olds. The initial intent-to-treat model included intervention conditions with time and the seasonal and COVID dummy variables at the observation level and city-level characteristics as covariates. The second model included intervention dosage level at the observation level along with the same covariates included in the initial model. Total motor vehicle crashes were included as an exposure variable to account for time-related variation and differences in the number of crashes between intervention and control cities. We conducted analyses to assess possible decay in intervention effects after March 2016 by excluding data during the intervention period from April 2013 through March 2016. Because of the monthly fluctuations in motor vehicle crashes, we also conducted sensitivity analyses with 6- and 12-month moving averages for both SVN and total crashes. Analyses were conducted in Stata Version 17.0 software (StataCorp LP, College Station, TX), allowing for random effects to account for variance attributable to the correlation of repeated observations nested within cities; robust standard errors were used to account for the nonnormal distribution of dependent variables.

We also estimated cost savings of the intervention based on alcohol-related crashes prevented during the postintervention period. This was based on cost estimates for alcohol-related motor vehicle crashes provided by the National Highway Traffic Safety Administration (Blincoe et al., 2023) and the costs of intervention implementation (U.S. $95,000 per intervention city per year × 4 years).

## Results

### Multi-level regression analyses

**Figure 1.**
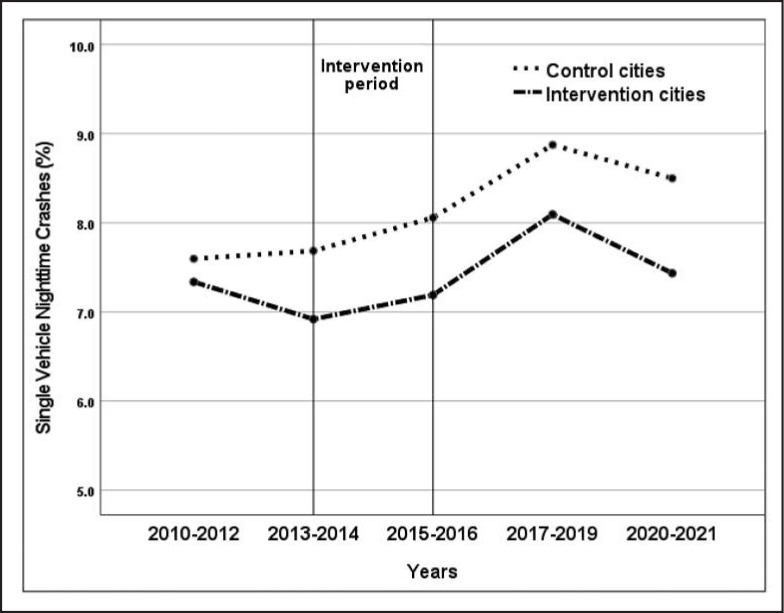
Trends in single-vehicle nighttime crashes as a percentage of total crashes from 2010 to 2021 by intervention condition. Values in the figure are predicted by intervention condition and other covariates in the regression model.

Results of multi-level negative binomial regression analyses are provided in Table 1. The initial intent-to-treat regression model for 15- to 30-year-olds indicated a significant reduction in the likelihood of SVN crashes in intervention cities relative to controls when controlling for the time trend, seasonal and COVID pandemic effects at the observation level, and city characteristics. The incidence rate ratio (IRR) indicates a 12% lower likelihood of SVN crashes among 15- to 30-year-old drivers in intervention cities from 2013 to 2021 relative to controls. The relative change in the percentage of SVN crashes predicted by the regression model is illustrated in Figure 1. Analyses did not indicate any significant intervention dosage effects on SVN crashes. Additional analyses conducted to assess possible decay in intervention effects indicated a comparable intervention effect after March 2016 (IRR [95% CI] = 0.86 [0.79, 0.94], *p* < .001). However, a regression model with data limited to the initial intervention period indicated no intervention effect on SVN crashes (IRR [95% CI] = 0.91 [0.81, 1.03], *p* = .14). Additional sensitivity analyses with 6- and 12-month moving averages for SVN and total motor vehicle crashes yielded results very similar to those obtained with actual monthly counts of crashes.

**Table 1.**
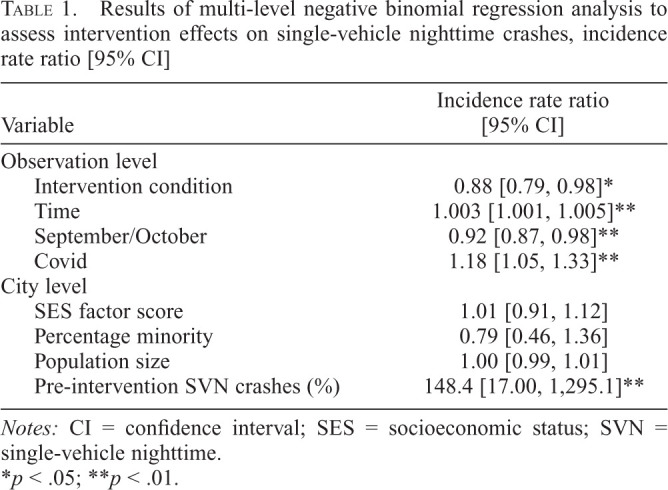
Results of multi-level negative binomial regression analysis to assess intervention effects on single-vehicle nighttime crashes, incidence rate ratio [95% CI]

Variable	Incidence rate ratio [95% CI]
Observation level	
Intervention condition	0.88 [0.79, 0.98]*
Time	1.003 [1.001, 1.005]**
September/October	0.92 [0.87, 0.98]**
Covid	1.18 [1.05, 1.33]**
City level	
SES factor score	1.01 [0.91, 1.12]
Percentage minority	0.79 [0.46, 1.36]
Population size	1.00 [0.99, 1.01]
Pre-intervention SVN crashes (%)	148.4 [17.00, 1,295.1]**

*Notes:* CI = confidence interval; SES = socioeconomic status; SVN = single-vehicle nighttime.

* *p* < .05;

** *p* < .01.

Based on the total number of SVN crashes among 15- to 30-year-olds in control cities during the postintervention period and the proportion of SVN/total crashes in intervention cities, there were approximately 700 fewer SVN crashes in intervention cities relative to controls. This includes approximately 12 fewer crashes with fatalities, 33 fewer crashes with severe injuries, and 200 fewer crashes with nonsevere injuries. We estimated a cost savings of U.S. $355.4 million.

## Discussion

The results suggest that the intervention reduced alcohol-related crashes beyond the life of the grant that supported it. In addition, the effect was sufficiently robust that factoring in the effects of the COVID pandemic and controls for preintervention differences in SVN across cities did not offset it. Being a population-level intervention, the difference amounts to significant numbers—700 fewer alcohol-related crashes and a savings of an estimated U.S. $355 million.

Here, we found no effect of dosage level. The original article found a surprisingly greater effect among cities that did not meet the target for implementation. The extended postintervention period seemed to eliminate that effect in the long run. This may mean that cities could achieve a reduction in alcohol-related crashes even when implementation is weak and/or sporadic. This bears further investigation, of course.

Although there does seem to be a continuing discontinuity between the intervention and control communities' alcohol-involved crash rates, the data here cannot provide a definitive explanation for the difference past the project period. Among the possibilities are that the difference is attributable to a form of inertia, whereby drinkers and drivers are behaving as though the implementation is still under way. Alternatively, it may be that community agencies, particularly law enforcement agencies, may have incorporated some aspects of the intervention into their routine in the years after the intervention period. This could happen if the intervention's emphasis on high visibility of enforcement established some channels of communication or dissemination that had not been used before. Finally, if less likely, the community agencies may have decided to continue the implementation activities even without the funding that had been available initially.

For all the reasons above, this report is not a study of sustainability per se. That would require original data collection across all 24 cities to record which, if any, of the intervention components remained in place after funding stopped. Nevertheless, by whatever mechanism, these findings give us reason to hope and expect that maintaining multi-component community interventions like this one may not be as challenging as many think. Research on sustainability is still uncommon but necessary to achieve progress in public health.

## References

[B1] Blincoe L. Miller T. Wang J.-S. Swedler D. Coughlin T. Lawrence B. Guo F. Klauer S. Dingus T. 2023 February The economic and societal impact of motor vehicle crashes, 2019 (Revised) DOT HS 813 403 https://crashstats.nhtsa.dot.gov/Api/Public/ViewPublication/813403

[B2] Saltz R. F. Paschall M. J. O'Hara S. E. 2021 Effects of a community-level intervention on alcohol-related motor vehicle crashes in California cities: A randomized trial American Journal of Preventive Medicine 60 1 38 46 10.1016/j.amepre.2020.08.009 33221142 PMC8492018

[B3] Voas R. B. Romano E. Peck R. 2009 Validity of surrogate measures of alcohol involvement when applied to nonfatal crashes Accident Analysis & Prevention 41 3 522 530 10.1016/j.aap.2009.02.004 19393802 PMC2776062

